# Morbid Appearances after Death, from Colica Pictonum

**Published:** 1829

**Authors:** E. J. Coxe


35. *Morbid appearances after death from Colica Pictonum.* By E. J. Coxe, M. D.

In the last Number of the North American Medical and Surgical Journal, Dr. Coxe has reported a fatal case of lead colic, with the postmortem examination. As this disease is becoming more frequent in the West, particularly in Cincinnati and other manufacturing towns; and as opportunities for autopsic inspection (happily) do not often occur, we shall lay before our readers, an account of the dissection of this case. The knife was wielded by Dr. Horner.

*Dissection.*—The abdominal muscles were drawn inwards towards the spine, and the parietes of the abdomen when struck indicated the presence of a smaller quantity of gas than usual in the hollow viscera.

The liver healthy, but of a yellowish tinge, the peri biliarii were occupied with bile, and had their parietes dyed by it. Gall-bladder empty and contracted, containing only a few drops of bile.

The spleen healthy, and about three inches in length.

Pancreas healthy.

The stomach contained a pint of gas. Its mucous coat was covered with a layer of viscid mucous, coloured yellow by bile. On removing this lamina, but little vascular injection was seen; a patch, an inch in diameter, of small ecchymosed spots, was seen near the lesser curvature; there were here and there at distant intervals, other small dots of blood, but the general complexion of the mucous coat was of a dingy or yellowish white, like old ivory. The mucous coat was generally so soft that it could be torn off by scraping with the thumb nail, and then came off in a pulpy state instead of in strips; but in the left extremity it was so soft as to suffer abrasion from much slighter causes than scratching with the nail. The duodenum and jejunum were internally of a yellow colour from the stain of their contents, which were bile and mucus mixed. The ileum was occupied by a small quantity of green purulent matter formed probably of the same substances, the colour of the bile having been altered perhaps by some acid fermentation or secretion there. The contents of the colon, which were merely enough to smear its surface, were the same as those of the jejunum.

The great intestine was collapsed, and in the highest possible state of irritation on its internal coat, which was studded with small tumours of a deep purple, or the colour of coagulated venous blood, from the quantity of blood deposited in the substance of this coat. This appearance was produced at the base of each of the rugæ or small folds of the latter, as if there had been a deposite of serum in the cellular coat which thickened it, and caused the folds to project in the form of pendulous tumours into the cavity of the gut. These tumours being blackened on the surface by the quantity of blood deposited as aforesaid in the substance of the mucous coat, gave to the gut the appearance of beginning mortification, as in the highest forms of dysentery. This condition of purple tumours prevailed universally at the caput coli, forming the entire surface there, and for some inches upwards; it then decreased gradually in the transverse colon and vanished in the sigmoid flexture.

The muciparous glands of the colon studded its whole surface where this violent hemorrhagic irritation prevailed; they were for the most part distant only two or three lines or less from one another, and projected into the cavity of the gut. Their size was for the most part about half a line, as it is in cholera infantum. I did not perceive any ulceration in them, the disease being perhaps in too early a stage for that. They were also conspicuous in the inferior six inches of the ileum. The peritoneal surface of all the abdominal viscera had a yellow tinge from the bile. The kidneys and bladder were healthy.

The semilunar ganglion of the sympathetic was white, firm and healthy. The viscera of the thorax were healthy, excepting some slight old pleuretic adhesions.—pp. 246, 248.

